# Factors Associated with Breastfeeding Initiation and Duration in Greece: Data from the Hellenic National Nutrition and Health Survey

**DOI:** 10.3390/children9111773

**Published:** 2022-11-18

**Authors:** Efthimia Spyrakou, Emmanuella Magriplis, Vassiliki Benetou, Antonis Zampelas

**Affiliations:** 1 Department of Hygiene, Epidemiology and Medical Statistics, School of Medicine, National and Kapodistrian University of Athens, 75 Mikras Asias Street, 115 27 Athens, Greece; 2 Department of Food Science and Human Nutrition, Agricultural University of Athens, Iera Odos 75, 118 55 Athens, Greece

**Keywords:** breastfeeding, initiation, duration, risk factors, determinants, caesarean section, prematurity, infant feeding, Greece

## Abstract

Despite its well-documented benefits, breastfeeding rates and duration worldwide do not meet the recommended goals set by the World Health Organization. Data on infant feeding, socio-demographic, lifestyle and perinatal factors were used from 490 mothers (and 958 children), participants at the Hellenic National Nutrition and Health Survey between 2013–2015. Clustered multiple logistic regression and multilevel mixed-model regression analyses were performed to identify factors associated with breastfeeding initiation and duration, respectively. Maternal lower education [Odds Ratio (OR): 2.29, 95% Confidence intervals (95% CI): 1.30–4.04; *p* = 0.004], smoking during pregnancy (OR: 3.08, 95% CI: 1.64–5.77; *p* < 0.001), caesarean section (OR = 3.26, 95% CI: 1.83–5.83; *p* < 0.001), prematurity (OR = 2.74, 95% CI: 1.40–5.37; *p* = 0.003) and higher birth order (OR = 1.30, 95% CI: 1.04–1.62; *p* = 0.020) were associated with increased odds of not initiating breastfeeding. Living in rural areas [beta coefficient *b* = −27.93, *p* = 0.043], smoking during pregnancy (*b* = −64.47, *p* < 0.001), caesarean section (*b* = −28.76, *p* = 0.046) and prematurity (*b* = −46.67, *p* = 0.048) were significantly associated with shorter breastfeeding duration. Children born chronologically closer to the survey were more likely to breastfeed and for longer periods. Educational promotion and enhancement of breastfeeding awareness that account for maternal exposures are required. Prevention of prematurity and avoidance of unnecessary caesarean section is crucial, while additional breastfeeding support is needed when preterm birth occurs, or caesarean section cannot be avoided.

## 1. Introduction

The short- and long-term benefits of breastfeeding for the child, the mother, the society and the environment have been well documented, with breastfeeding being considered one of the most important elements for the promotion of public health and an investment for the health of future generations [1]. Nevertheless, worldwide breastfeeding rates, especially those of exclusive breastfeeding, are far from complying to global public health recommendations and nutrition goals set by the World Health Organization (WHO); the later aiming for at least 50% of six-month-old infants worldwide to be exclusively breastfed by 2025 [2,3,4]. Europe, in total, has the lowest breastfeeding rates compared to other continents with only 25% of infants between 2006–2012 being exclusively breastfed at 6 months of age compared to 43% in the WHO South-East Asia Region [2,5]. In Greece, data from the latest nationally representative study conducted during 2017, showed that 94% of mothers initiated breastfeeding after birth and that 80%, 56% and 45% of the infants were breastfed by the end of the 1st, 4th and 6th completed month of life, respectively [6]. Nevertheless, less than 1% of infants were exclusively breastfed at the end of six months. 

Breastfeeding rates greatly vary between countries due to differences in several factors identified as determinants of breastfeeding initiation, duration and exclusivity, such as factors related to the social and cultural context of the country, the healthcare system and the related health professionals, the cost, mother’s beliefs and health status, as well as the support of the social environment [7,8,9,10,11]. The perinatal period seems to be very important for breastfeeding initiation and duration. Smoking during pregnancy, gaining excessive weight during pregnancy and delivering by caesarean section have all been negatively associated with breastfeeding [12,13,14]. Gestational diabetes mellitus, premature birth and high parity may also unfavourably affect breastfeeding success [15,16,17]. 

Based on the most recent studies conducted in Greece, a variety of factors have been identified as barriers of breastfeeding initiation and/or duration, among them, lower maternal education [6,12], caesarean section [6,18], smoking during pregnancy [12,13,18], and low birth weight [6]. Maternity hospital practices, such as prescription of infant formula and not providing rooming-in for breastfed infants, have also been reported as negative determinants of breastfeeding initiation and duration [6,19]. On the other hand, maternal higher education, previous breastfeeding experience, skin-to-skin contact and education and encouragement to breastfeed, are some of the factors that have been positively associated with breastfeeding initiation and/or duration [6,12,19].

Identifying factors that influence breastfeeding initiation and duration, especially those that negatively affect them, in specific local situations and settings is extremely important for the successful development and implementation of national infant feeding policies and action plans for the protection, promotion and support of breastfeeding at the local and national level [11]. Moreover, although plenty of evidence exists on breastfeeding determinants, some of them, such as maternal education, act in different directions depending on the local situations, thus providing one further reason why research on this area is worth undertaking in different populations across the world. 

Based on the above, we have explored factors that may be associated with breastfeeding initiation and duration using data from a sample of mothers living in Greece, participants at the Hellenic National Nutrition and Health Survey (HNNHS). 

## 2. Materials and Methods

### 2.1. Study Design 

Data were derived from the HNNHS, a population-based, cross-sectional survey conducted from September 2013 through May 2015 in Greece. HNNHS aimed to assess health and nutritional status of the Greek population, including children over 6-month-old and adults. Exclusion criteria at baseline included individuals that did not speak Greek, were pregnant or lactating, members of the armed forces, individuals that resided in institutions (e.g., nursing homes) and those that were unable to provide informed consent due to any cause unless a first relative was able to assist in the process. A random stratified design was implemented based on the 2011 census data which was used to achieve representativeness in six age groups (0–19 years, 20–65 years, and +65 years), in females and males across three geographical regions of Greece (main Metropolitan areas, islands and mainland). More details related to the description of HNNHS, aim, population characteristics and design can be found elsewhere [20]. 

### 2.2. Participants 

For the purposes of this study, all female participants of HNNHS with at least one child and available information on infant feeding were eligible. Thus, data from a total of 490 women (24.6% primiparous) and their children, 958 children overall, were included in this study. The study was conducted in accordance with the Helsinki Declaration and was approved by the Ethics Committee of the Department of Food Science and Human Nutrition of the Agricultural University of Athens and by the Hellenic Data Protection Authority (HDPA). All members of the staff signed confidentiality agreements and all adult volunteers signed an informed consent form.

### 2.3. Data Collection

All questionnaires used in HNNHS were based on a priori knowledge and constructed from components of previously validated questionnaires [20]. An initial interview by trained personnel obtaining information on anthropometric, socio-demographic and lifestyle parameters took place at the volunteer’s house, with the use of a specially designed computer software, namely the Computer Assisted Personal Interview (CAPI), in order to minimize response biases and misclassification (minimize volunteer burden and maximize reliability of collected data). Information on maternal socio-demographic characteristics, such as area of residence, age, years of education, occupation and marital status were recorded. 

### 2.4. Information on Infant Feeding and Gestation/Childbirth Characteristics

Female participants were asked to report for each child whether breastfeeding was initiated (yes/no) as well as the duration of breastfeeding. The initiation of breastfeeding was considered positive if the mother answered that she had breastfed her child, irrespective of the point in time. Thus, initiation of breastfeeding was equivalent to breastfeeding the infant at any point in time. With respect to duration of breastfeeding, in order to help the mother to report it more accurately, mothers were given the option to report it in days or months, either on average or within a range (between—to). Breastfeeding was defined as any breastfeeding, meaning the infant feeding practice where the infant receives breast milk and any other food or liquid including non-human milk and formula, which is in line with WHO definitions [21]. Perinatal information for each pregnancy and child, such as information on gestational age, birth weight, type of delivery and history of smoking during pregnancy were also recorded. Children with birth weight less than 2.500 g were characterized as low birth weight, whereas preterm birth was defined as any birth before 37 completed weeks of gestation based on WHO definition. 

### 2.5. Statistical Analysis

Frequency tables and percentages were used to describe categorical and ordinal variables and medians and percentiles for breastfeeding duration. As two or more children could have the same mother, we used clustered analysis to the mothers when calculating effect estimates, to account for within mother variation at each birth. This assured that maternal age at birth (mother’s age at each delivery), which was calculated using mother’s birth date and the child’s birth date of every child, was accounted for as well. 

Clustered multiple logistic regression was used to examine the odds of not initiating breastfeeding with specific variables. The choice of these variables was based on a priori knowledge regarding the factors that have been associated with breastfeeding initiation and duration from the literature, as well as significant differences observed from descriptive statistics between breastfed and non-breastfed children. Based on the analysis chosen (clustered by mothers ID), the variability within maternal age for each biological child is accounted for, and by including years since birth between individual variation is controlled for as well. 

Years that passed since birth in relation to the time of recruitment in the study, were calculated to evaluate possible changes in practices and policies related to the protection and support of breastfeeding across time, something we wanted to account for. Of note, to test that no residual confounding from maternal age at birth affected model estimates, maternal age was included in the model when building it, but no significant effect and no change in the estimates of the other variables between breastfed and non-breastfed children was observed. Maternal education (more than 13 years vs. 13 years and less), maternal area of residence (main metropolitan areas vs. other areas-including islands and mainland), maternal smoking during pregnancy (yes vs. no), type of delivery (caesarean section vs. normal delivery), birth order (per increment of one child), birth weight (continuously, per 500 g), and years since birth (per one decade increment and more) were all introduced in the model. Because preterm birth (yes vs. no) was highly correlated with birth weight they were alternatively introduced in the model. Goodness-of-fit tests using Hosmer–Lemeshow test (HL-test) were also calculated for the multiple logistic regression models using preterm birth, and alternatively birth weight, in the final models. The HL-test was better with preterm birth than with birth weight, and thus we proceeded with preterm birth in the main model for breastfeeding duration as well. 

Multilevel mixed-model regression analysis compared to multiple regression analysis, was used to examine the possible associations of the above-mentioned factors with breastfeeding duration, clustering by maternal ID. An interaction term was introduced in the model for preterm birth and caesarean section, as well as, in the alternative model for birth weight and caesarean section, in order to account for possible interaction between the specific variables (included since it modified results and Likelihood Ratio (LR) test increased). The statistical significance level was set to be 0.05. Analyses were performed using STATA 13.1 (STATA Corporation, College Station, TX, USA).

## 3. Results

From the 958 children, a total of 829 (86.5%) were breastfed, whereas among the breastfed, the median duration of breastfeeding was 120 days (25th percentile: 42 days, 75th percentile: 180 days). 

Table 1 presents the distribution of maternal socio-demographic characteristics and lifestyle habits during pregnancy, as well as perinatal characteristics among the 958 children by breastfeeding initiation status. More children whose mothers had higher educational level or were living in the main metropolitan areas were breastfed, while less children whose mothers smoked during pregnancy were breastfed. Children with low birth weight (<2.500 g) compared to normal and high birth weight (>4000 g), and premature children compared to full term, had lower rates of breastfeeding. Moreover, caesarean section was more frequent among children who were not breastfed in comparison to those who were breastfed. As the order of birth was increasing, there was a tendency to not be breastfed, though the findings were not statistically significant. More women with recent birth seemed to have breastfed their children compared to those who had delivered their children in previous years (Table 1). 

Adjusted odds ratios and 95% confidence intervals derived from the multiple clustered logistic regression investigating the odds of not initiating breastfeeding (equivalent to odds of not being breastfed) with maternal and lifestyle parameters and child’s perinatal characteristics are presented in Table 2. Children with mothers of lower educational level had at least twice higher odds of not being breastfed compared to children with mothers of higher educational level (OR = 2.29, 95% CI: 1.30–4.04; *p* = 0.004). Similarly, children whose mothers were smoking during their pregnancy had more than three times the odds of not being breastfed (OR = 3.08, 95% CI: 1.64–5.77; *p* < 0.001), compared to children whose mothers were not smoking during pregnancy. Children who were delivered with caesarean section were at least 3 times more likely to not being breastfed in comparison to children born with vaginal labour (OR = 3.26, 95% CI: 1.83–5.83; *p* < 0.001). Premature infants had almost three times higher odds of not being breastfed (OR = 2.74, 95% CI: 1.40–5.37, *p* = 0.003). When birth weight was alternatively introduced in the model (instead of prematurity), for every 500-g increment in birth weight, the odds of not breastfeeding decreased by 26% (OR = 0.74, 95% CI: 0.56–0.99; *p* = 0.040). On the other hand, for every additional birth, the odds of not being breastfed increased by 30% (OR = 1.30, 95% CI: 1.04–1.62; *p* = 0.020). Finally, for every decade that has passed since birth, the odds of not being breastfed increased by 46% (OR = 1.46, 95% CI: 1.11–1.94; *p* = 0.010). 

In Table 3, the results of the multilevel mixed-model regression analysis for breastfeeding duration among the 824 breastfed children with maternal sociodemographic and lifestyle characteristics and perinatal factors, as well as the median duration (and 25th and 75th percentiles) of breastfeeding (in days) for each characteristic/factor, are displayed. Children with mothers who smoked during their pregnancy or mothers who were living in rural, non-metropolitan areas, were breastfed for shorter periods (*b* = −64.47, *p* ≤ 0.001 and *b* = −27.93, *p* = 0.043, respectively). Moreover, children born with caesarean section were breastfed for shorter period in relation to those born normally (*b* = −28.76, *p* = 0.046), while children born prematurely were also breastfed for shorter periods (*b* = −46.67, *p* = 0.048). When birth weight was introduced alternatively to prematurity in the model, increased birth weight, per 500 g, was associated with increased duration of breastfeeding, although this result did not reach statistical significance (*b* = 5.53, *p* = 0.162). Finally, children who were born in past years compared to those who were born in more recent (in relation to the recruitment) years had shorter duration of breastfeeding (*b* = −30.57, *p* < 0.001).

## 4. Discussion

In this sample of women living in Greece, we found evidence that lower maternal education, maternal smoking during pregnancy, caesarean section, prematurity, lower birth weight, and higher birth order were all negatively associated with breastfeeding initiation. Caesarean section, maternal smoking during pregnancy, preterm birth and living in rural areas were found to be associated with shorter breastfeeding duration. Time since birth was associated with breastfeeding initiation and duration showing that children born in more recent to recruitment years were more likely to breastfeeding and for longer periods compared to children born in previous periods. 

Lower maternal educational level has been consistently associated with lower breastfeeding rates, especially in studies conducted among women living in economically developed countries [11,22]. Mothers with lower educational levels are less likely to have the skills to identify official and valid infant feeding recommendations, the ability to keep up with updated breastfeeding information, to follow their healthcare provider’s advice and to receive proper prenatal care, compared to more educated mothers [23,24]. Nevertheless, in some societies, the opposite association has also been reported, a finding that can be partially attributed to differences in the working and cultural environment of women with high educational level, among which, the short maternity leave, the heavy workload, as well as the ability to afford purchasing imported infant formula [25]. 

Caesarean section was negatively associated with breastfeeding initiation and duration compared to vaginal delivery. Caesarean section, planned or not, has been associated with delayed breastfeeding initiation, poor first breastfeeding attempt results, lower rates of exclusive breastfeeding and early breastfeeding cessation [26,27,28,29,30,31]. Possible reasons that could explain these findings are linked with maternal post-operational pain and use of anaesthesia which affect maternal consciousness and delay breastfeeding initiation. Lactogenesis may also be disrupted in women delivering with a caesarean section due to the decrease in oxytocin secretion or maternal stress which both may lead to decreased milk production [22]. Inhibition of infant’s sucking reflex and disruption of mother-infant interaction are additional possible mechanisms [30]. Furthermore, women with planned caesarean section are less likely to intend to breastfeed compared to women who had a vaginal birth or an emergency caesarean delivery [29]. Initiation of breastfeeding within the first hour of life, according to WHO recommendations, is crucial for the establishment of lactation and breastfeeding success [32]. Supporting mothers during the first hours and days after caesarean section is of great importance, since early breastfeeding behaviours seem to be more important than the influence of the operation per se [29].

Preterm birth was negatively associated with breastfeeding initiation. This is a consistent finding and prematurity is considered one of the major negative predictors of breastfeeding initiation [33,34,35]. Preterm infants may not be able to feed at the breast at birth, but they can receive the benefits of human milk immediately and breastfeed eventually [35]. It is worth mentioning also, that medical complications of prematurity, various neonatal comorbidities and admission to neonatal intensive care unit are crucial factors influencing breastfeeding initiation in preterm infants [36]. Fan et al. reported that there was no association of prematurity with breastfeeding rates one-month post-partum in healthy, without complications preterm infants with no admission to intensive care unit [37]. Unfortunately, no information on the medical history of preterm infants was available in this study to evaluate this association. Preterm birth was also associated with shorter breastfeeding duration. Flacking et al. studied 37,343 mothers of 2093 preterm and 35,250 term infants in Sweden and found that mothers of preterm infants had higher risk of weaning before two, four, six and nine months after delivery compared to mothers with full-term infants and highlighted the importance of breastfeeding support after hospital discharge in preterm infants [38]. 

Lower birth weight, when alternatively introduced to prematurity, due to their high correlation, was also negatively associated with breastfeeding initiation. An analysis using birth cohorts from the National Health and Nutrition Examination Survey in USA, reported that low birth weight infants had lower percentages of breastfeeding initiation and duration compared to the normal weight ones [33]. Similarly with preterm infants, breastfeeding of low birthweight infants presents several challenges due to maternal and infant physiology, psychology, and their environment [35]. Among others, mothers of low-birth-weight infants often worry about the adequacy of their milk supply and they ultimately introduce unwarranted infant feeding formulas [39]. Currently, WHO guidelines and implementation guidance, state that all infants, including small, sick and/or preterm infants, should be fed human milk [35]. In fact, human milk is even more beneficial for the health of preterm infants compared to full-term infants, as well as for low-birth-weight infants compared to normal weight and supporting mothers to establish and maintain milk supply and preparing the health care system to provide an overall family-centred care within a supportive environment is crucial [35]. 

Higher birth order was associated with lower odds of breastfeeding in this study, similar with previous studies [40,41,42]. Multiparous mothers may have more reasons to stop breastfeeding compared to primiparous, such as heavier family obligations, less anxiety to prove their motherly skills and more attention needed for older children [16,40]. Nevertheless, this association may depend on previous infant feeding experiences as well [16]. Thus, mothers with a previous negative breastfeeding experience are less likely to initiate breastfeeding in the next birth and the opposite. Although higher birth order was associated with lower odds of breastfeeding, no association between birth order and breastfeeding duration was found in this study.

According to the latest European Perinatal Health Report [43], prevalence of smoking during gestation is above 10% in several European countries. In Greece, it is reported that prevalence of smoking during pregnancy ranges between 17–26% [44,45], while in a recent meta-analysis prevalence ranged between 15–20% [46]. In our study, almost 13% of the participants reported smoking during pregnancy. Smoking is considered one of the most established negative predictors of breastfeeding initiation and duration [13,22,47]. It is estimated that women who smoked during their pregnancies have three times higher odds of never initiating breastfeeding, or if they had initiated breastfeeding, they breastfeed their infants for shorter time durations compared to none-smokers [22,47]. Shorter breastfeeding duration may be attributed to the reduced amount of breast milk produced by mothers who smoke [47]. Nicotine that passes in breast milk can reduce prolactin and eventually its volume. Moreover, smoking during pregnancy and lactation can alter the composition of human milk and its health promoting properties, by reducing its content in total lipids, changing its immune status and reducing its antioxidant properties [48].

Although no association was evident between the area of residence and breastfeeding initiation, women living in rural areas were found to breastfeed for shorter duration compared to those living in the main metropolitan areas. One possible explanation is that rural areas, in general, have less advanced primary health care facilities compared to the main metropolitan areas, which are also less accessible, something relevant for several places in Greece [49]. This finding however should be interpreted with caution since the area of residence reported by the mother at baseline may be different than the one at the time of pregnancy and lactation. 

The current study also found that infants born chronologically closer to the survey, had higher odds of initiating breastfeeding and were breastfeeding for longer duration compared to infants born chronologically far from the survey. This observation coincides with the study by Iliodromiti et al. [6]. in which any breastfeeding and exclusive breastfeeding rates were higher in the second national cross-sectional study conducted in Greece during 2017 compared to the first one conducted ten years prior, in 2007. The widely implementation of national breastfeeding promoting programs and the introduction of new policies and legislation for the protection of mother’s and infant’s rights, as well as the support and strengthening of the Baby Friendly Hospital Initiative in Greece could provide an explanation for this observation. 

Our study has several limitations. All information about breastfeeding initiation and duration, as well as other past medical history and lifestyle factors during pregnancy, were self-reported. Thus, a degree of recall bias has been introduced due to the retrospective nature of the information leading to either under-or-over reporting of breastfeeding initiation and duration, especially if time since the previous lactation was long. A degree of residual confounding is also possible since information on other potentially important factors, such as mother’s body mass index, child’s medical conditions and previous experience of breastfeeding were not available. Furthermore, no adequate data on type of breastfeeding, and especially exclusive breastfeeding, were available in order to examine factors related to exclusive breastfeeding initiation and duration. The cross-sectional nature on the study does not permit to make causal inferences in most of the associations observed. It should also be noted that the final sample of this study is, most probably, not representative of the Greek population of mothers although associations found could be generalized to women with similar sociodemographic characteristics. Nevertheless, information in this study was obtained with the help of trained personnel and recorded in previously validated questionnaires following an established research protocol. 

In conclusion, several socio-demographic and lifestyle factors of the mother as well as perinatal factors of the infant, were associated with breastfeeding initiation and duration in this study sample. Mothers with lower educational level, mothers who smoked during pregnancy, mothers who delivered with caesarean section, mothers with infants with lower birth weight or premature infants, and those residing in rural areas require special attention and support. Moreover, considering that the prevalence of caesarean deliveries is increasing globally [31], and in Greece is unreasonably high [50], every effort should be made to avoid unnecessary caesarean sections and reduce their frequency. Moreover, as prematurity is still the leading cause of death among children younger than 5-years-old [51], promoting breastfeeding, and the use and availability of human milk, among preterm infants, will substantially help to improve their survival. Initiatives such as the Baby Friendly Hospital Initiative should expand to all maternity hospitals in the country featuring a baby and mother-friendly maternity environment. Reliable information and education of the expectant and new parents, and the whole society, on the benefits of breastfeeding, in line with national infant feeding policies and recommendations, should be ensured. Despite the increasing trends in breastfeeding indicators observed in Greece during the last decade (2007–2017), partly due to the implementation of relevant supportive policies and initiatives at the national level, [6] still more efforts are required. A comprehensive national action plan for the protection, promotion and support of breastfeeding integrating, among others, multilevel interventions before, during and after pregnancy and considering the needs and characteristics of women and families least likely to breastfeed, should be in place for a sufficient length of time in order to reach the recommended target for breastfeeding and ultimately achieve better health for infants and future adults.

## Figures and Tables

**Table 1:** Distribution of maternal sociodemographic characteristics and lifestyle habits during pregnancy and perinatal characteristics among 958 children by breastfeeding initiation status.

Variables	No Breastfeeding		Breastfeeding		*p*-Value ^1^
	*N* = 129	%	*N* = 829	%	
Maternal education (in years)					<0.001
<13	89	69.0	395	47.6	
≥13	40	31.0	434	52.4	
Area of residence					0.050
Main metropolitan areas	68	52.7	512	61.8	
Other areas	61	47.3	317	38.2	
Maternal smoking during pregnancy					<0.001
Yes	33	25.6	91	11.0	
No	96	74.4	738	89.0	
Birthweight (in grams)					<0.001
<2500	24	18.6	33	4.0	
2500–3499	70	54.3	539	65.0	
3500–3999	26	20.2	197	23.8	
≥4000	9	7.0	60	7.2	
Preterm birth					<0.001
Yes	22	17.0	54	6.5	
No	107	83.0	775	93.5	
Type of delivery					<0.001
Caesarean section	56	43.4	204	24.6	
Normal birth	73	56.6	625	75.4	
Birth order					0.080
1st	55	42.6	435	52.5	
2nd	57	44.2	312	37.6	
3rd	16	12.4	65	7.8	
≥4th	1	0.8	17	2.1	
Years since birth					0.01
<10	17	13.2	233	28.1	
10–19	39	30.2	193	23.3	
20–29	46	35.7	257	31.0	
30+	27	20.9	146	17.6	

^1^*p*-values derived from Chi-square test.

**Table 2:** Clustered multiple logistic-regression-derived Odds Ratios (ORs) and 95% Confidence intervals (95% CI) assessing the odds of not initiating breastfeeding with maternal sociodemographic and lifestyle characteristics and perinatal factors.

Variable	Increment/Category	ORs	95% CI	*p*-Value
Maternal education	<13 years ≥13 years	2.29 baseline	1.30–4.04	0.004
Area of residence	Other areas Main Metropolitan areas	1.53 baseline	0.88–2.64	0.130
Maternal smoking during pregnancy	Yes No	3.08 baseline	1.64–5.77	<0.001
Type of delivery	Caesarean section Normal birth	3.26 baseline	1.83–5.83	<0.001
Preterm birth	Yes No	2.74 baseline	1.40–5.37	0.003
Birth order	For each child increase	1.30	1.04–1.62	0.020
Years since birth	For each decade increase	1.46	1.11–1.94	0.010
*Goodness-of-fit HL-test p = 0.883*				
*Alternative introduced variable to prematurity*				
Birth weight	Per 500 g	0.74	0.56–0.99	0.040
*Goodness-of-fit HL-test p = 0.515*				

Goodness-of-fit HL-test: Goodness-of-fit Hosmer–Lemeshow test.

**Table 3:** Multilevel mixed-model regression-analysis-derived coefficients (*b*) and 95% Confidence Intervals (95% CI) assessing the association of maternal sociodemographic and lifestyle characteristics and perinatal factors with breastfeeding duration.

Variables	Median Breastfeeding Duration	25th, 75th Percentile	B Coefficient	95% CI	*p*-Value
Maternal education					
<13 years	90	40, 180	−1.72	−28.81, 25.37	0.900
≥13 years	120	60, 210	baseline		
Area of residence					
Main Metropolitan areas	120	40,210	baseline		
Other areas	90	45, 180	−27.93	−54.96, −0.90	0.043
Maternal smoking during pregnancy					
Yes	60	30, 120	−64.47	−101.29, 27.65	<0.001
No	120	60, 210	baseline		
Type of delivery					
Caesarean section	120	45, 210	−28.76	−57.01, −0.51	0.046
Normal birth	120	42, 180	baseline		
Preterm birth					
Yes	60	35, 120	−46.67		
No	120	50, 210	baseline	−92.84, −0.49	0.048
Birth order					
1st	90	40, 180			
2nd	120	56, 195			
3rd	150	60, 210			
4th+	150	90, 300			
For each child increase			−4.02	−14.74, 6.69	0.462
Years since birth					
<10	180	60, 270			
10–19	90	60, 180			
20–29	90	40, 180			
30+	90	40, 180			
Per decade increase			−30.57	−42.47, −18.67	<0.001
*Alternative introduced variable to prematurity*					
Birth weight					
<2500	60	30, 120			
2500–2999	90	40, 180			
3000–3499	120	40, 180			
3500–3999	120	60, 240			
4000+	120	60, 210			
Per 500 gr increment			5.53	−3.43, 20.50	0.162

An interaction term was also introduced in the model between preterm birth and mode of delivery.

## Data Availability

The data presented in this study are available on request from the corresponding author. The data are not publicly available because the database includes multiple measurements and exposure variables that are not part of this study, and ethical issues apply. “MDPI Research Data Policies” at https://www.mdpi.com/ethics (accessed on 1 October 2022, 18:28.).

## References

[1] Victora C.G., Bahl R., Barros A.J.D., França G.V., Horton S., Krasevec J., Murch S., Sankar M.J., Walker N., Lancet Breastfeeding Series Group (2016). Breastfeeding in the 21st Century: Epidemiology, Mechanisms, and Lifelong Effect. Lancet.

[2] Bagci Bosi A.T., Eriksen K.G., Sobko T., Wijnhoven T.M., Breda J. (2015). Breastfeeding Practices and Policies in WHO European Region Member States. Public Health Nutr..

[3] Cattaneo A., Yngve A., Koletzko B., Guzman L.R. (2005). Protection, Promotion and Support of Breast-Feeding in Europe: Current Situation. Public Health Nutr..

[4] World Health Organization (2014). Global Nutrition Targets 2025: Policy Brief Series. https://www.who.int/publications/i/item/WHO-NMH-NHD-14.2.

[5] Theurich M.A., Davanzo R., Busck-Rasmussen M., Díaz-Gómez N.M., Brennan C., Kylberg E., Bærug A., McHugh L., Weikert C., Abraham K. (2019). Breastfeeding Rates and Programs in Europe: A Survey of 11 National Breastfeeding Committees and Representatives. J. Pediatr. Gastroenterol. Nutr..

[6] Iliodromiti Z., Zografaki I., Papamichail D., Stavrou T., Gaki E., Ekizoglou C., Nteka E., Mavrika P., Zidropoulos S., Panagiotopoulos T. (2020). Increase of Breast-Feeding in the Past Decade in Greece, but Still Low Uptake: Cross-Sectional Studies in 2007 and 2017. Public Health Nutr..

[7] Rollins N.C., Bhandari N., Hajeebhoy N., Horton S., Lutter C.K., Martines J.C., Piwoz E.G., Richter L.M., Victora C.G., Lancet Breastfeeding Series Group (2016). Why Invest, and What It Will Take to Improve Breastfeeding Practices?. Lancet.

[8] Mahesh P.K.B., Gunathunga M.W., Arnold S.M., Jayasinghe C., Pathirana S., Makarim M.F., Manawadu P.M., Senanayake S.J. (2018). Effectiveness of Targeting Fathers for Breastfeeding Promotion: Systematic Review and Meta-Analysis. BMC Public Health.

[9] Wallenborn J.T., Wheeler D.C., Lu J., Masho S.W. (2019). Importance of Familial Opinions on Breastfeeding Practices: Differences between Father, Mother, and Mother-In-Law. Breastfeed. Med..

[10] Pérez-Escamilla R., Martinez J.L., Segura-Pérez S. (2016). Impact of the Baby-Friendly Hospital Initiative on Breastfeeding and Child Health Outcomes: A Systematic Review. Matern. Child Nutr..

[11] EU Project on Promotion of Breastfeeding in Europe (2008). Protection, Promotion and Support of Breastfeeding in Europe: A Blueprint for Action (Revised).

[12] Tavoulari E.-F., Benetou V., Vlastarakos P.V., Psaltopoulou T., Chrousos G., Kreatsas G., Gryparis A., Linos A. (2016). Factors Affecting Breastfeeding Duration in Greece: What Is Important?. World J. Clin. Pediatr..

[13] Vassilaki M., Chatzi L., Bagkeris E., Papadopoulou E., Karachaliou M., Koutis A., Philalithis A., Kogevinas M. (2012). Smoking and Caesarean Deliveries: Major Negative Predictors for Breastfeeding in the Mother-Child Cohort in Crete, Greece (Rhea Study). Matern. Child Nutr..

[14] Martin H., Thevenet-Morrison K., Dozier A. (2020). Maternal Pre-Pregnancy Body Mass Index, Gestational Weight Gain and Breastfeeding Outcomes: A Cross-Sectional Analysis. BMC Pregnancy Childbirth.

[15] Laine M.K., Kautiainen H., Gissler M., Pennanen P., Eriksson J.G. (2021). Impact of Gestational Diabetes Mellitus on the Duration of Breastfeeding in Primiparous Women: An Observational Cohort Study. Int. Breastfeed. J..

[16] Buckman C., Diaz A.L., Tumin D., Bear K. (2020). Parity and the Association between Maternal Sociodemographic Characteristics and Breastfeeding. Breastfeed. Med..

[17] Byerly T., Buckman C., Tumin D., Bear K. (2020). Prematurity and Breastfeeding Initiation: A Sibling Analysis. Acta Paediatr..

[18] Benetou V., Tavoulari E.F., Gryparis A., Linos A. (2019). Reducing Caesarean sections and smoking after delivery could help to tackle shorter exclusive breastfeeding duration. Acta Paediatr..

[19] Tavoulari E.F., Benetou V., Vlastarakos P.V., Andriopoulou E., Kreatsas G., Linos A. (2015). Factors affecting breast-feeding initiation in Greece: What is important?. Midwifery.

[20] Magriplis E., Dimakopoulos I., Karageorgou D., Mitsopoulou A.V., Bakogianni I., Micha R., Michas G., Ntouroupi T., Tsaniklidou S.M., Argyri K. (2019). Aims, Design and Preliminary Findings of the Hellenic National Nutrition and Health Survey (HNNHS). BMC Med. Res. Methodol..

[21] World Health Organization (2008). Indicators for assessing infant and young child feeding practices. Proceedings of the Conclusions of a Consensus Meeting.

[22] Cohen S.S., Alexander D.D., Krebs N.F., Young B.E., Cabana M.D., Erdmann P., Hays N.P., Bezold C.P., Levin-Sparenberg E., Turini M. (2018). Factors Associated with Breastfeeding Initiation and Continuation: A Meta-Analysis. J. Pediatr..

[23] Skafida V. (2009). The Relative Importance of Social Class and Maternal Education for Breast-Feeding Initiation. Public Health Nutr..

[24] Pang W.W., Aris I.M., Fok D., Soh S.E., Chua M.C., Lim S.B., Saw S.M., Kwek K., Gluckman P.D., Godfrey K.M. (2015). Determinants of Breastfeeding Practices and Success in a Multi-Ethnic Asian Population. Birth.

[25] Zhao J., Zhao Y., Du M., Lee A.H. (2017). Maternal Education and Breastfeeding Practices in China: A Systematic Review and Meta-Analysis. Midwifery.

[26] Raihana S., Alam A., Huda T.M., Dibley M.J. (2021). Factors Associated with Delayed Initiation of Breastfeeding in Health Facilities: Secondary Analysis of Bangladesh Demographic and Health Survey 2014. Int. Breastfeed. J..

[27] Paksoy Erbaydar N., Erbaydar T. (2020). Relationship between Caesarean Section and Breastfeeding: Evidence from the 2013 Turkey Demographic and Health Survey. BMC Pregnancy Childbirth.

[28] Zhang F., Cheng J., Yan S., Wu H., Bai T. (2019). Early Feeding Behaviors and Breastfeeding Outcomes after Cesarean Section. Breastfeed. Med..

[29] Hobbs A.J., Mannion C.A., McDonald S.W., Brockway M., Tough S.C. (2016). The Impact of Caesarean Section on Breastfeeding Initiation, Duration and Difficulties in the First Four Months Postpartum. BMC Pregnancy Childbirth.

[30] Prior E., Santhakumaran S., Gale C., Philipps L.H., Modi N., Hyde M.J. (2012). Breastfeeding after Cesarean Delivery: A Systematic Review and Meta-Analysis of World Literature. Am. J. Clin. Nutr..

[31] Boerma T., Ronsmans C., Melesse D.Y., Barros A.J., Barros F.C., Juan L., Moller A.B., Say L., Hosseinpoor A.R., Yi M. (2018). Global Epidemiology of Use of and Disparities in Caesarean Sections. Lancet.

[32] World Health Organization (2003). Global Strategy for Infant and Young Child Feeding.

[33] Herrick K.A., Rossen L.M., Kit B.K., Wang C.Y., Ogden C.L. (2016). Trends in Breastfeeding Initiation and Duration by Birth Weight among US Children, 1999–2012. JAMA Pediatr..

[34] Callen J., Pinelli J. (2005). A review of the Literature examining the benefits and challenges, incidence and duration, and barriers to breastfeeding in preterm infants. Adv. Neonatal Care.

[35] World Health Organization, The United Nations Children’s Fund (UNICEF) (2020). Protecting, Promoting and Supporting Breastfeeding: The Baby-Friendly Hospital Initiative for Small, Sick and Preterm Newborns.

[36] Ericson J., Eriksson M., Hoddinott P., Hellström-Westas L., Flacking R. (2018). Breastfeeding and Risk for Ceasing in Mothers of Preterm Infants—Long-Term Follow-Up. Matern. Child Nutr..

[37] Fan H.S.L., Wong J.Y.H., Fong D.Y.T., Lok K.Y.W., Tarrant M. (2019). Breastfeeding Outcomes among Early-Term and Full-Term Infants. Midwifery.

[38] Flacking R., Nyqvist K.H., Ewald U. (2007). Effects of Socioeconomic Status on Breastfeeding Duration in Mothers of Preterm and Term Infants. Eur. J. Public Health.

[39] Agyekum M.W., Codjoe S.N.A., Dake F.A.A., Abu M. (2022). Is Infant Birth Weight and Mothers Perceived Birth Size Associated with the Practice of Exclusive Breastfeeding in Ghana?. PLoS ONE.

[40] Lok K.Y.W., Bai D.L., Tarrant M. (2015). Predictors of Breastfeeding Initiation in Hong Kong and Mainland China Born Mothers. BMC Pregnancy Childbirth.

[41] Bailey B.A., Wright H.N. (2010). Breastfeeding Initiation in a Rural Sample: Predictive Factors and the Role of Smoking. J. Hum. Lact..

[42] Fang Z., Liu Y., Wang H., Tang K.T. (2019). The Patterns and Social Determinants of Breastfeeding in 12 Selected Regions in China: A Population-Based Cross-Sectional Study. J. Hum. Lact..

[43] Euro-Peristat project (2018). European Perinatal Health Report. Core Indicators of the Health and Care of Pregnant Women and Babies in Europe in 2015. http://www.europeristat.com/index.php/reports/european-perinatal-health-report-2015.

[44] Vardavas C.I., Patelarou E., Chatzi L., Roumeliotaki T., Sarri K., Murphy S., Koutis A., Kafatos A.G., Kogevinas M. (2010). Factors Associated with Active Smoking, Quitting, and Secondhand Smoke Exposure among Pregnant Women in Greece. J. Epidemiol..

[45] Vivilaki V.G., Diamanti A., Tzeli M., Patelarou E., Bick D., Papadakis S., Lykeridou K., Katsaounou P. (2016). Exposure to Active and Passive Smoking among Greek Pregnant Women. Tob. Induc. Dis..

[46] Lange S., Probst C., Rehm J., Popova S. (2018). National, Regional, and Global Prevalence of Smoking during Pregnancy in the General Population: A Systematic Review and Meta-Analysis. Lancet Glob. Health.

[47] Timur Taşhan S., Hotun Sahin N., Omaç Sönmez M. (2016). Maternal Smoking and Newborn Sex, Birth Weight and Breastfeeding: A Population-Based Study. J. Matern.-Fetal Neonatal Med..

[48] Nordhagen L.S., Kreyberg I., Bains K.E.S., Carlsen K.H., Glavin K., Skjerven H.O., Småstuen M.C., Hilde K., Nordlund B., Vettukattil R. (2020). Maternal Use of Nicotine Products and Breastfeeding 3 Months Postpartum. Acta Paediatr..

[49] Mariolis A., Mihas C., Alevizos A., Mariolis-Sapsakos T., Marayiannis K., Papathanasiou M., Gizlis V., Karanasios D., Merkouris B. (2008). Comparison of Primary Health Care Services between Urban and Rural Settings after the Introduction of the First Urban Health Centre in Vyronas, Greece. BMC Health Serv. Res..

[50] Antoniou E., Orovou E., Iliadou M., Sarella A., Palaska E., Sarantaki A., Iatrakis G., Dagla M. (2021). Factors Associated with the Type of Cesarean Section in Greece and Their Correlation with International Guidelines. Acta Inform. Med..

[51] Chawanpaiboon S., Vogel J.P., Moller A.B., Lumbiganon P., Petzold M., Hogan D., Landoulsi S., Jampathong N., Kongwattanakul K., Laopaiboon M. (2019). Global, regional, and national estimates of levels of preterm birth in 2014: A systematic review and modelling analysis. Lancet Glob. Health.

